# Inherent paradoxes in the shift to autonomous solutions provision: a multilevel investigation of the shipping industry

**DOI:** 10.1007/s11628-021-00458-5

**Published:** 2021-09-18

**Authors:** Håkon Osland Sandvik, David Sjödin, Thomas Brekke, Vinit Parida

**Affiliations:** 1 Department of Business, History and Social Sciences, University of South-Eastern Norway, Raveien 215, 3184 Borre, Norway; 2 Entrepreneurship and Innovation, Luleå University of Technology, 97187 Lulea, Sweden

**Keywords:** Digital servitization, Paradoxes, Autonomous solutions, Digitalization, Multilevel framework

## Abstract

Transforming a traditional industry by adopting autonomous solutions is complex, generating paradoxical tensions on multiple aggregate levels. We undertake an in-depth case study of a leading maritime autonomous solutions provider and its ecosystem partners. We apply the paradox lens using thematic analysis. Our research contributes to the digital servitization literature by identifying six paradoxes inherent in the shift to autonomous solutions, nested in the micro, meso, and macro levels. We develop a multilevel framework of organizational paradoxes, delineating cascading effects of paradoxes across levels. We offer valuable insights for providers to integrate multilevel perspectives into the shift to autonomous solutions.

## Introduction

Digitalization has begun the process of transforming many traditional industries. The economic landscape is being changed worldwide through the leveraging of tremendous efficiency improvements derived from digital technologies such as AI, automation, and analytics (Tronvoll et al. 2020; Gaiardelli et al. 2021; Sjödin et al. 2021). According to an 2018 IDC report, 38% of traditional businesses had already adopted a digital strategy, and the IMF estimates that 65% of the world’s GDP will be digitalized by 2022 (FinancesOnline 2020). These technological shifts have been further accelerated in response to Covid-19 and are expected to have long-term effects as we move to the “next normal” (Rapaccini et al. 2020; Zeng et al. 2020). While consumers use online activity to cope with social distancing measures and for knowledge acquisition (López-Cabarcos et al. 2020; Xie et al. 2020), companies are learning to adapt to the crisis through collaborative knowledge creation (Al-Omoush et al. 2020) and by making changes to their business models that may well prove to be beneficial in a post-pandemic world (Kraus et al. 2020). Our data—collected between December 2019 and March 2021—suggest that despite certain challenges, this trend has the potential to spill over into the shipping industry as autonomous solutions reduce human contact.

Innovative technology providers work to digitalize their industries through a digital servitization process, defined as “the transition toward smart product-service-software systems that enable value creation and capture through monitoring, control, optimization, and autonomous function” (Kohtamäki et al. 2019, p. 383). Arguably, the most disruptive of these shifts is the transition to autonomous solutions (Porter and Heppelmann 2014). For example, technology providers in traditional industries such as the shipping industry work to transform their industry toward adoption of autonomous solutions—a shift that will profoundly change the industry’s traditional business models, as autonomous vessels enable new services like remote operations which open the door for changed ownership structures and selling technological solutions through outcome-based contracts (Munim 2019). However, despite various promising initiatives, the shift to large-scale autonomous solutions has been impeded in many industrial domains.

A key challenge facing traditional industries that are engaged in this transformation to autonomous solutions is the persistent tensions between the established ways of doing business and embracing new business models (Thomson et al. 2021). For example, technology providers face internal tensions between developing customized solutions and maintaining efficient standardization (Kohtamäki et al. 2020). Furthermore, as digitalization entails distributed innovation space and agency (Nambisan et al. 2017), the shift to autonomous solutions may extend beyond the firm, involving persistent tensions at the ecosystem and market levels that make transitioning even more complicated. For example, our data suggest that for seafarers, the simultaneous prospect of increased job attractiveness at lower autonomy levels collides with the potential for job losses at higher levels of autonomy. A second maritime case in point is the contradiction between the opportunity provided by autonomous solutions to reduce the risk of human error and the societal barrier of perceived safety standards being lowered. Thus, we argue that identifying and understanding the paradoxical tensions between opportunities and barriers related to offering autonomous solutions call for a multilevel analysis at firm, ecosystem, and society levels.

From a theoretical standpoint, this study seeks to bring nuance to dichotomous studies that have highlighted either the benefits or the barriers of autonomous solutions by bringing the two together through the paradox lens (Munim 2019; Ghaderi 2019; Rødseth 2017; Ringbom 2019). Often in organizational life, leaders face difficulties in simply choosing between alternatives. In such situations, the paradox lens may bring theoretical clarity to these issues by revealing their true nature and explaining their interrelatedness, persistence, and incongruity (Smith and Lewis 2011). Adopting a paradoxical perspective may add necessary shading in coming to understand the shift to autonomous solutions in an industrial setting. We have identified several knowledge gaps in this domain:

Firstly, there is a need to identify and describe the paradoxes that companies face as they move from product provider to autonomous solutions provider (Kohtamäki et al. 2020). Indeed, prior studies have mainly discussed opportunities and barriers to the transition without considering their paradoxical nature (Paiola and Gebauer 2020; Munim 2019). As various autonomous products have been commercialized for consumer markets, marketing scholars have already begun to dissect the effect of autonomous products on consumers (Leung et al. 2018; Rijsdijk and Hultink 2003; de Bellis and Johar 2020; Chiang and Trimi 2020). Similarly, as autonomous solutions have the potential to transform industrial markets, scholars have started to investigate the link between technology, business models, and ecosystems (Thomson et al. 2021). In a B2B setting, manufacturers may benefit from a digital servitization strategy in the move to providing autonomous services. As manufacturers continue to experience paradoxical tensions in seeking to combine service logic with product logic, scholarly attention has turned to the paradoxes inherent in servitization, although external paradoxes have not yet been investigated (Kohtamäki et al. 2020).

Secondly, given the complexity in making this transition, a multilevel investigation of nested clusters of paradoxes is clearly needed at the micro, meso, and macro levels. No business is an island: leading an industrial transformation means instigating change at the industry and ecosystem levels (Kamalaldin et al. 2021; Sklyar et al. 2019; Nambisan et al. 2017). We argue that the specific setting of the shipping industry, as it moves to transformative autonomous solutions, is particularly fruitful terrain to identify multilevel paradoxes given the industry’s central role in globalization and the inherent interrelatedness between the ecosystem actors (Kim et al. 2021). Additionally, considering the barriers, benefits, and complexities that this highly traditional industry faces in transitioning to autonomy (Ghaderi 2019; Ringbom 2019; Munim 2019), we hypothesize that initiating a transformation on such an industry-wide scale is not likely to be achieved without encountering paradoxical relationships. Furthermore, it should be noted that vital interaction between stakeholders takes place on different aggregate levels: it is likely, therefore, that such paradoxical tensions will emerge in the encounter with multiple levels. Indeed, leading the industrial digital servitization process implies complex interaction with actors on different aggregate levels—namely, employees, companies, markets, and society at large. Yet, prior studies in digitalization and servitization lack insights into organizing and understanding transformational paradoxes using a multilevel framework (Smith and Beretta 2021; Kohtamäki et al. 2020).

We developed the following research questions to address these research gaps: how do paradoxes emerge in the industrial shift to autonomous solutions, and how do they manifest themselves on the micro, meso, and macro levels? Accordingly, this study seeks to identify and describe paradoxes technology providers encounter in their digital servitization process toward autonomous solutions. To address this objective, we build on an in-depth case study of a leading autonomous solutions provider and its extended ecosystem of partners. Our data are derived from 29 interviews with senior managers exercising different business functions. In our analysis of the data, we employ a thematic analysis approach (Braun and Clarke 2006).

Our analysis identifies six paradoxes nested in the micro (technology development, organizational identity), meso (coopetition ecosystem, ecosystem evolution), and macro (regulatory and policy, customer interaction) levels. Accordingly, the present study reveals how an autonomous solutions provider experiences the underlying tensions. In essence, we provide an empirical account from the shipping industry of how a company experiences its leading role in an industrial digital servitization process that is geared to providing autonomous solutions.

We contribute to the literature on digital servitization and associated paradoxes in several ways. Firstly, we provide a coherent map of paradoxes inherent in the digital servitization process of moving toward autonomous solution provision. We identify six paradoxes of which four are new and two corroborate findings already present in the servitization literature. Consequently, the discussion of barriers and benefits of maritime autonomous solutions is suitably enriched. Secondly, we contribute by offering a multilevel framework that presents an analysis of the paradoxes inherent in autonomous solutions provision and their interlinks. This study is, to the best of our knowledge, the first to investigate servitization paradoxes in a multilevel framework. Such external perspectives have been called for by prior research in digitalization and servitization (Smith and Beretta 2021; Kohtamäki et al. 2020). Finally, we contribute by providing a descriptive account of paradoxes intrinsic to a traditional industry’s shift to autonomous solutions and showing how paradoxes can affect each other through cascading effect across levels.

This paper is structured as follows: in the literature review section, the shift to digital servitization and autonomous solution is discussed before presenting the multilevel paradox framework. Section 3 outlines the study methods. In the result section, we present six paradoxes and discuss interrelations between them. Finally, contributions, limitations, and suggestions for future research are offered in Sect. 5.

## Review of literature

### The shift to digital servitization and autonomous solutions

Whereas the digitalization literature emphasizes digital technology as an enabler to business model innovation at the organizational and ecosystem level (Parida et al. 2019; Linde et al. 2021), the servitization literature concerns such change in business models for manufacturing firms moving from product to service dominant logic (Baines et al. 2009; Hyun and Kim 2021). Although digital technologies have been an essential ingredient of servitization from its conception, the two literature streams have only recently converged explicitly as digital servitization (Gebauer et al. 2021).

In practice, several industries have transformed through digital servitization, enabling manufacturing companies to provide advanced services (Cenamor et al. 2017). Wärtsilä, Caterpillar, and Rolls Royce are prominent examples of large industrial companies that have successfully implemented digital servitization by leveraging sensor and software technology forming product–service–software systems (PSSS) (Kohtamäki et al. 2019).

Autonomous solutions are essentially product–service–software systems (PSSS) that can function independently of human supervision (Thomson et al. 2021). Conceptually, autonomous solutions have been placed on the supreme end of a digitalization spectrum, reflecting digital technology features ranging from monitoring to control, optimization, and autonomy (Porter and Heppelmann 2014). As such, the transition toward autonomous solution is seen as the most advanced form of digital servitization (Kohtamäki et al. 2019) and is arguably the most disruptive, potentially reshaping traditional industry boundaries (Porter and Heppelmann 2014). As autonomous solutions allow for reduced human intervention, they may yield significant economic, environmental, and social benefits depending on their industrial application (Parida et al. 2019; Porter and Heppelmann 2014). However, barriers exist as regulations still require humans to operate systems in many domains (Hussain and Zeadally 2019; Ringbom 2019; Porter and Heppelmann 2014; Paiola and Gebauer 2020). Additionally, as autonomous solutions allow technology providers to take more responsibility of customers’ operational processes, customer maturation can be a substantial barrier to this advanced form of digital servitization (Lerch and Gotsch 2015). Similarly, as digitalization generally raises the need for collaboration across firm boundaries (Kamalaldin et al. 2021; Sklyar et al. 2019; Parida et al. 2019; Nambisan et al. 2017), the development of suitable business models that ensures alignment of ecosystem partners as well as customers may be key to overcoming these barriers (Thomson et al. 2021).

We follow Adner (2017)’s conceptualization of ecosystem-as-structure which puts the value proposition center stage, placing emphasis on the activities and interdependencies between ecosystem actors, and leaves room for single firms to take part in multiple ecosystems simultaneously and even to adopt different ecosystem roles. Following from the empirical context, the focal value proposition of this study is *maritime autonomous solutions*. This broad value proposition includes products and services that deliver capabilities for surface ships at various degrees of autonomy. As a rule of thumb, both the benefits of and the barriers to autonomous solutions increase along with degree of autonomy. Following from the empirical context of this study, our informants referred primarily to the international maritime organization’s (IMO) four degrees of autonomy for maritime surface ships: degree one is a ship with automated processes and decision support; degree two is a remotely controlled ship with seafarers onboard; degree three is a remotely controlled ship without seafarers onboard; and degree four is a fully autonomous ship.

### Toward autonomous shipping

A company in the shipping industry is “a commercial firm that is active in either ship owning, trading, operations, and/or commercial and technical innovations, however, as an industry it is the overall value chain that matters” (Lorange 2009, p. 3). Albeit services have long been an intrinsic part of the shipping value chain, however in digitalizing the industry toward autonomous solutions, technology providers are required to undergo a servitization process changing their business model from sales of products and after sales services, to providing autonomous solutions with continuous services embedded in the solutions (Munim 2019).

Although the shipping industry is gradually adopting innovative digital products that improve maneuvering, navigation, and planning, and its business operations have remained largely traditional since the dawn of containers in 1956, which paved the way for modern shipping (Ghaderi 2019; Levinson 2016). Now, however, maritime technology providers along with their ecosystem partners are launching initiatives to fully digitalize the shipping industry by commercializing autonomous solutions for surface vessels. Autonomous solutions have lurked in the background of shipping ever since autonomous underwater vehicles (AUV) were commercialized a decade ago. So far, autonomous surface vessels have followed a similar path to AUV technology, starting with research and development of pilot programs and working their way to commercialization (Ghaderi 2019).

It is the short sea shipping segment that is best placed to benefit the most with the introduction of continuously unmanned vessels (CUS), monitored or controlled by a shore control center (SCC) (Munim 2019; Ghaderi 2019). In this segment, crew salary constitutes a major part of the operational expenses in developed countries; thus, autonomous solutions allowing crew reduction may help cut operational expenses drastically (Munim 2019; Ghaderi 2019). Also, when considering the regulatory barriers to crew reduction, overcoming them is considered a much less daunting task within a flag state than between flag states (Ghaderi 2019). It is therefore likely that the short sea shipping segment will be the first to widely adopt high-degree autonomous solutions.

Whereas the benefits of autonomous solutions represent the entrepreneurial opportunity, their barriers represent the entrepreneurial risk. These barriers and benefits are opposing sides of the same coin intrinsic to the digital servitization process toward autonomy. In essence, they can be seen as the originating sources of the opposing elements that induce paradoxical tensions. In the following section, we explain the theoretical paradox lens and introduce a multilevel analysis framework.

### Understanding multilevel paradoxes in the transition to autonomous solutions

Paradoxes have amused thinkers since ancient times. Today scholars study paradoxes in many varied fields ranging from philosophy and physics to the social sciences. Transaction cost economics and classical organizational theory such as contingency theory offer perspectives that help to clarify problems and assist decision makers in arriving at the best possible option among opposing alternatives (Donaldson 2001; Williamson 1979). While both strategic and short-term objectives may be achieved by choosing one alternative, it often involves sacrificing another. Organizational life is, however, full of tensions and inconsistencies that leaders cannot simply choose away—for example, employees’ goals (higher salary) and company’s goals (higher profit). The paradox lens is an alternative way of framing problems that can help decision makers to develop coping strategies so that they can “live with” the tensions rather than devise strategies that force them to choose between alternatives.

Smith and Lewis (2011, p. 386) define a paradox as “*contradictory yet interrelated elements that exist simultaneously and persist over time*.” Thus, we identify a paradox with reference to three conditions:

1. There must be underlying tensions whose elements are individually coherent and logical but inconsistent or incompatible when juxtaposed. Contradictory.
2. An element cannot be chosen over the other, but companies must embrace both elements simultaneously. Interrelated.
3. The tensions must be long-term and non-trivial. Persistent.

The paradox lens brings richness and depth to the sometimes overly simplistic framing of dilemmas and dialectics. Dilemmas frame tensions between two competing alternatives with clear advantages and disadvantages, implying a choice needs to be made between the alternatives. Dialectics, on the other hand, are when the contradictory elements creating the tensions (thesis and antithesis) cannot be solved by choosing between them but the situation is resolved by adopting a third integrated alternative (synthesis) (Smith and Lewis 2011). Theoretically, there are clear distinction between paradoxes, dilemmas, and dialectics. In practice, dilemmas and dialectics may resurface if the choice or integration proves temporary, making them paradoxical. In such situations, companies could benefit from reframing these issues as paradoxes and working toward coping strategies rather than opting for choice or integration.

Since digital servitization processes are inherently complex, there is a need to incorporate ecosystem and market perspectives into paradox analysis. For this purpose, we adapt a multilevel framework open eco-innovation distinguishing between micro, meso, and macro levels (Garcia et al. 2019; Chistov et al. 2021; Markard and Truffer 2008). Similar to previous studies in service innovation (Teixeira et al. 2012; Baron et al. 2018; Patrıcio et al. 2011), we apply the multilevel analysis framework from the perspective of firms as they encounter these different levels. Acknowledging the existence of different variations to what constitutes each level (Kiefer et al. 2021; Frow et al. 2014; Ghazinoory et al. 2020), we emphasize the need to distinguish between paradoxes that are internal to the organization, emerging at the collaborative level between organizations and in the organizations encounter with societal levels (Garcia et al. 2019; Chistov et al. 2021). Because paradoxes are intrinsically complicated, this distinction is important as it provides clear boundaries from the technology providers’ perspective, without adding complexity. At the micro level, our analysis focuses on aspects internal to the organization including interactions among individuals and between divisions. The meso level concerns the organization as an entity in relation to other organizations in the ecosystem. The highest aggregate level is the macro level, which is concerned with society at large, including governmental bodies and markets. Our analysis, therefore, reflects the paradoxes that technology providers encounter when interacting with stakeholders at the different levels.

**Fig. 1:**
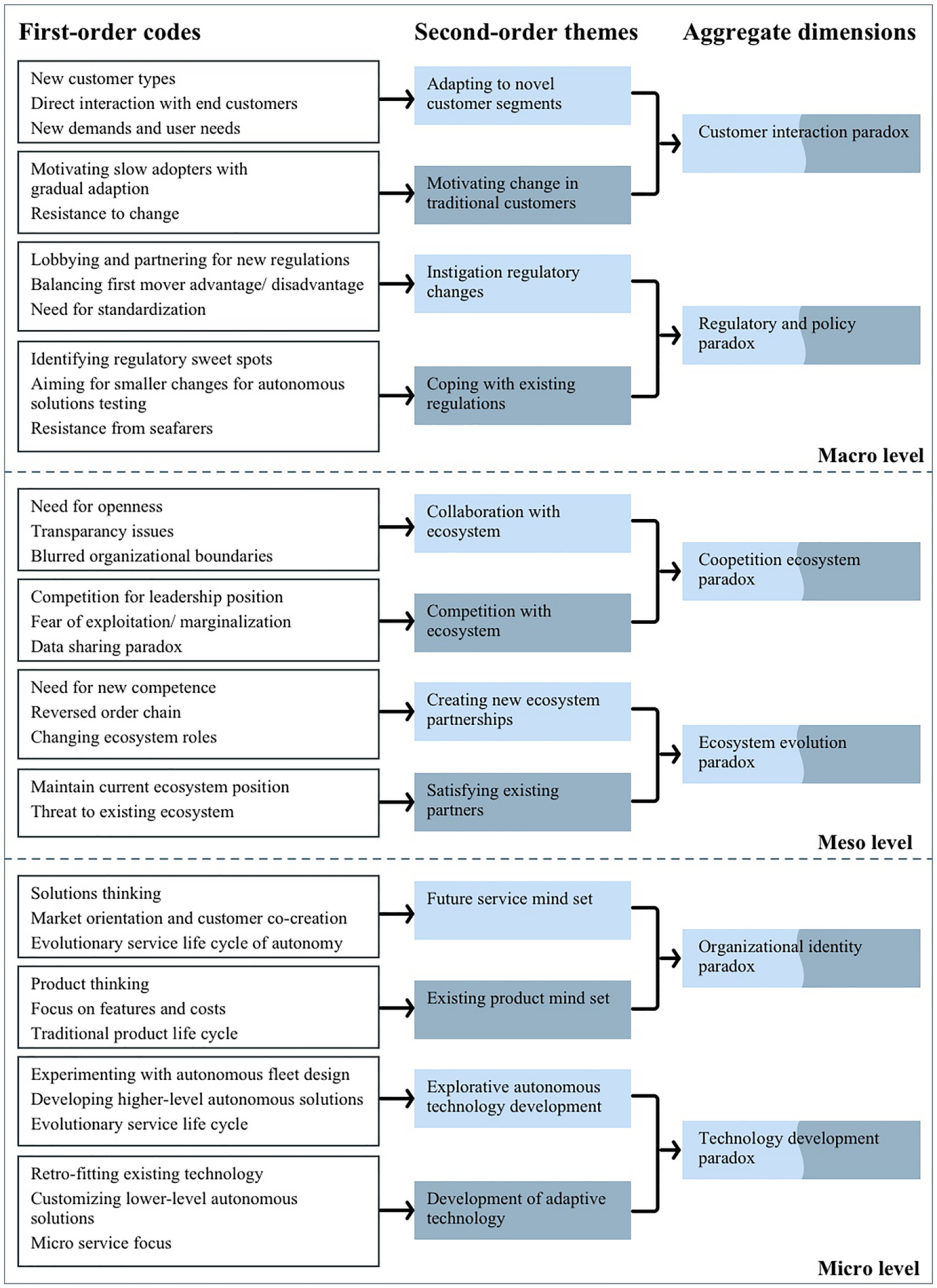
Data structure

Additionally complicating, paradoxical tensions can be interwoven (Sheep et al. 2017), nested across space and time (Jarzabkowski et al. 2013), and cascading across levels (Andriopoulos and Lewis 2009; Smith and Lewis 2011). This means that events on the macro level can influence paradoxes on the meso and micro levels and vice versa (Garcia et al. 2019). For instance, the archetype paradoxes of belonging, and organizing and performing intersect via tensions between the individual and the aggregate (Jarzabkowski et al. 2013; Smith and Lewis 2011). Albeit our data structure (Fig. 1) is presented static (for clarity), our analysis reveals the existence of nested relationships between the levels in the data. In Sect. 4.4, we “*zoom out*” to investigate relationships between the paradoxes (Schad and Bansal 2018, p. 1498). Before presenting the paradoxes in the result section, we outline the research strategy applied, followed by our case selection, data collection, and analysis.

## Methods

### Research strategy

We used an explorative in-depth case study approach to conduct the research. Case studies are a good way to investigate contemporary events (Yin 2014) because they richly describe the context in which the events occur and deliver deep insights into dynamic social structures (Dyer and Wilkins 1991). Therefore, the in-depth case study approach suits our explorative purpose very well, considering the contemporality and dynamic structure of the industry-wide digital transformation context and the complex social structures underpinning organizational and inter-organizational phenomena such as paradoxes.

### Case selection

In selecting our case, we applied a theoretical sampling strategy (Corbin and Strauss 2015). Given the industry’s history, position, and trajectory, we believed that transforming shipping industry by shifting to autonomous solutions would be a particularly fruitful venture for identifying multilevel paradoxes. Consequently, this study includes data from three companies in the shipping industry, chosen for their roles in the shift to maritime autonomous solutions. Following the in-depth case study approach (Dyer and Wilkins 1991), focused on one technology-providing company (Shipcontrol) working to transform the shipping industry toward adopting autonomous solutions. Furthermore, we included two of its closest partners, increasing the validity of external paradoxes. The focal company (Shipcontrol) and its two partners (Autosolution and Marinecompany) are separate business units that form part of the same ownership structure. This is contextually important because it affords them a high degree of coordination as they work to transform the industry, while simultaneously exercising their freedom to pursue individual business goals. We chose to focus on Shipcontrol because its large and complex organization with global reach and leading role in the maritime autonomous solutions-ecosystem would likely provide us with the in-depth data necessary to understand paradoxes of digital servitization toward autonomous solutions. To strengthen the multilevel analysis with external perspectives, we decided to include Autosolution and Marinecompany to the case. The number of respondents from each company reflects company size and their relative position in driving the digital servitization process toward autonomous solutions. Basic information of the case is presented in Table 1.

**Table 1:** Case and data information

Company	Sales/Employees	Solution	Respondent title	No. of interviews
Shipcontrol	M$ 730/3270	Autonomous solutions ranging from monitoring to fully autonomous	VP business development	1
			Product director	2
			Department manager	1
			Sales director	2
			Technical director	1
			VP business concepts	2
			General manager digital product	1
			Site manager remote operations	3
			Portfolio manager	3
			VP digital product services	1
			Manager Shipcontrol	2
			Manager marketing and sales	1
			Manager business development digital products	2
			Senior VP digital products	1
Autosolution	M$ 0/2	Services for remote and autonomous vessels	Chairman	1
			Business development manager	2(1)
			Manager marketing and sales	1(1)
Marinecompany	M$ 11/54	Operational management	General manager	1
			TOTAL	29

### Data collection

To collect rich descriptive data on paradoxes and their constitutive elements, a series of interviews were conducted in the case companies. Interviews are a well-suited method to obtain insights into how phenomena emerge (Yin 2014). The interviews were part of a broader overarching research program investigating the industrial transition to autonomous solutions. Thus, the process of analysis leveraged as desirable the disjuncture between interview questions and the questions guiding the coding and analysis of the data (Braun and Clarke 2006). Additionally, increasing reliability data from other publicly available sources such as company websites, annual reports, and industry podcasts were included for triangulation purposes (Miles et al. 2014). The data were collected over a 15-month period from December 2019 to March 2021. Having been collected just before and during the pandemic, the data provided unique insights into how contingencies affected the work of companies as they transition to autonomous solutions provision.

A total of 29 interviews were conducted, using a semi-structured approach with open-ended questions on various aspects of the autonomy transformation process. This allowed respondents to elaborate freely and add personal anecdotes to discussion topics that were especially important to them. The interviews were recorded and transcribed for analysis purposes. Notes were taken during the interviews. The data collection process was ended when theoretical saturation was reached (Corbin and Strauss 2015), i.e., when no new themes of paradoxical tensions emerged from data, and the paradoxical tensions identified where themselves were explored sufficiently in-depth to be theoretically developed. The respondents were senior managers handling different functions within their respective organizations. To ensure anonymity, acronyms of company names and positions were used. Table 1 displays information about the data collection.

### Data analysis

A thematic analysis was chosen because it is a method that can provide a rich, detailed, and complex account of the data, and it is capable of describing patterns across large qualitative datasets (Braun and Clarke 2006). This method aligns well with our research mission to identify paradoxical tensions and describe how these manifest themselves at the micro, meso, and macro levels. In conducting the analysis, we followed a process similar to the steps delineated by Braun and Clarke (2006). The analysis involved a series of iterations going back and forth between the steps to identify themes in the dataset. As a preliminary to the data analysis, we consulted the paradox literature and archetypes of organizational paradoxes to form the basis for the coding process (Smith and Lewis 2011).

As a first step in this highly iterative process, we immersed ourselves in the data by simultaneously listening to recordings and reading the transcripts. We were, therefore, able to pick up on verbal nuances and note respondents’ emphasis on different topics, thereby increasing the accuracy of the process (Braun and Clarke 2006). As we read and re-read the transcriptions, we searched for meanings and patterns to generate ideas for subsequent labeling and structures, taking notes and highlighting interesting passages. Based on the ideas and segments identified in the familiarization step, initial codes were generated to capture interesting elements in the raw data. Next, we grouped first-order codes into potential second-order themes and then refined them by consulting the literature to make sure codes were assigned to themes that corresponded to the appropriate level in the multilevel framework (micro, meso, macro). The themes were named and then reviewed to ensure internal homogeneity and external heterogeneity (Patton 1990). Finally, the second-order themes were collapsed into aggregate dimensions. This was undertaken in line with paradox theory. The aggregate dimensions reflect paradoxes generated by patterns among the second-order themes that correspond to opposite sides of the tensions identified (Smith and Lewis 2011). To increase reliability, the analysis was conducted by several researchers. Disagreements were resolved through iterative cycles of discussion guided by the paradox and digital servitization literature (Miles et al. 2014). At each cycles of discussion, the researchers reviewed each other’s suggestions to change, move, and/or remove codes, until converging on a single coherent data structure (Miles et al. 2014). After a series of iterative discussions, we were left with six paradoxes whose elements satisfactorily met the conditions of interrelatedness, contradiction, and persistence. As part of the procedure, a thematic map (Fig. 1) was constructed, portraying the data structure derived from the analysis.

## Results

In the findings section, we structure the identified paradoxes according to the aggregate level in which they occur. We start by explaining the micro-level paradoxes, we then investigate the paradoxes at the meso level, and, lastly, we describe the macro-level paradoxes. For each paradox, we first explain the paradoxical relationships in its various elements before zooming in and describing the substance of each element individually.

### Micro-level organizational paradoxes in the transition to autonomous solutions

The micro level focuses on the internal paradoxes of firms engaged in the industrial transition to autonomous solutions. We identify two key paradoxes on this level: the technology development paradox and the organizational identity paradox. These are explained in detail in the following paragraphs.

#### Technology development paradox

Firstly, we find that the shift to autonomous solutions provision creates a ***technology development paradox***. This paradox emerges from the tensions between *explorative autonomous technology development*—arising from the prospects and demands of a newbuilt autonomous fleet—and the *development of adaptive technology* focused on adding autonomous capabilities to the existing fleet. For technology providers, the two opposing technology development perspectives are paradoxical. On the one hand, major investment in explorative technology development is necessary in order to display the economic viability of unmanned vessels to ship owners and to showcase safety to authorities, pushing legislation for crew reduction. On the other hand, developing adaptive technology to suit the needs of the existing fleet is necessary to keep the autonomy project profitable and to create a ladder to climb for traditional customers who are digitalizing existing fleet. These two perspectives are interrelated. Without explorative autonomous technology development, technology providers would not be able to unlock the real potential benefits of maritime autonomy. Without the development of adaptive technology, the technology providers would not be able to bring along the ship owners who control the existing fleet, which is necessary for scalability and profitability. The two perspectives are contradictory because they draw from the same resource pool and because the solutions to some extent compete. Finally, the tensions will be present so long as the industry is in transition. Thus, while working to transform the industry, technology providers cannot choose between the explorative or adaptive technology development perspectives, but instead they must embrace both. Indeed, evidence of the technology development paradox appeared several times throughout our case studies. For example, the product director at Shipcontrol reiterated this need for forced ambidexterity in technology development:

“…We were aiming for the level 4 according to IMO, continuously unmanned operations; that was our goal, and we were working toward that. As we matured along the way, and the regulatory bodies matured along the way, we could see that it is a marathon, not a sprint, and we need to make money along the way.” (Product director, Shipcontrol).

The two technology development perspectives relate to opposite ends of the autonomy scale. *Explorative autonomous technology development* focuses on enabling a high level of autonomous capability to facilitate crew reduction, lower operational expenses, and shift the focus from mere technology development to vessel design. Inherent in explorative autonomous technology development is a holistic system perspective, which emphasizes end functionality and promotes experimental fleet design to take full advantage of the opportunities afforded by autonomy. This means developers need to maintain a broader oversight over the complete array of solutions and their integrations and not just the autonomous products referred to by Shipcontrol’s technical director:

“…you have an autonomous carrier moving the containers on land for the crane to load on the vessel, and the crane is automatic. In order to do this efficiently, there has to be a tight connection between the vessel, the crane, and the landside, even into the production systems of the factory. So, the way I see it, it is more the internal part of the whole chain that will influence the offerings, the services, and the systems.” (Technical director, Shipcontrol).

In contrast, technology providers need to pursue an exploitative perspective focused on the *development of adaptive technology* producing retrofit, autonomous solutions ready for sale in today’s existing fleet market. Because of heterogeneous technological maturity and the conditions within the existing fleet, retrofitting to deliver full autonomous capabilities involves extensive customization and, therefore, raises capital expenditure. Thus, retrofitting solutions for an existing fleet is usually restricted to lower-level autonomous capabilities such as monitoring and optimization technology. The exploitative perspective of adaptive technology development therefore leads technology providers to develop standardized autonomy product–service–software systems for autonomous micro-services. Shipcontrol’s product director explained how the exploitation of the existing market led to the development of lower-degree autonomous micro-services:

“We do see that the majority of the customers rely on the existing fleet, meaning they are looking toward performance optimization and safety optimization or reduction of the crew, but not complete removal of the crew” (Product director, Shipcontrol).

To sum up, technology providers experience the technology development paradox as tensions between differing perspectives of explorative and adaptive solution development. The tensions are paradoxical because, despite drawing from the same resource pool, technology providers need to pursue both approaches simultaneously for the foreseeable future, until the ecosystems, markets, and regulations have either transitioned or rejected autonomous solutions.

#### Organizational identity paradox

Secondly, we find that the shift to autonomous solutions provision creates an ***organizational identity paradox***. This paradox emerges from conflicting tensions between the increasing need to adopt a *future service mindset* through which autonomy is commercialized, while preserving the *existing product mindset* on which the technology-providing company is founded. Because autonomous solutions simultaneously increase the technological complexity and elevate the need to understand the customer's business, autonomy providers must embrace both product and service mindsets. Though contradictory, one cannot be chosen over the other; thus, the tensions are paradoxical. Informants emphasized how they struggled to incorporate the future service mindset into the existing product mindset permeating their organization:

“…the main challenge for a business like us, we are used to delivering projects, but we are not used to delivering services. So, we are looking into tools and philosophies, going from delivering a project to delivering a service, it demands a different focus from us. We are still working in the old-fashioned way, with purchases going to customers, invoicing them, and so on. There is a lot of evolution in this business today, with digitalization and being a more services-based operation actually.” (VP digital product services, Shipcontrol).

As touched upon by Shipcontrol’s manager of digital product services, the traditional existing product mindset is characterized by one-time monetary transactions with fixed delivery time frames and the explicit transfer of product ownership. Product thinking was evident in many of the autonomy providers’ activities. For instance, in technology development, product thinking manifests itself as a narrow focus on developing features while maintaining costs. In marketing, the product mindset involves searching for markets where the products can be sold rather than creating solutions that reflect market needs. The existing product mindset was deeply embedded in Shipcontrol’s culture and, therefore, integrating the future service mindset was still very much a work in progress for the case company.

“…yes, but we are working constantly with this to mature the whole organization, because with seven thousand seven hundred people, things take time.” (VP digital product services, Shipcontrol).

*The evolutionary service life cycle of autonomy* with continuous service delivery, continuous digital updates, and recurring revenue models stands in stark contrast to the temporally discrete *traditional product life cycles*, requiring the autonomy provider to shift to a service mindset. In contrast to the inside-out logic of the existing product mindset, the *future service mindset* emphasizes an outside-in market-orientated logic, where internal processes are guided by the customers. Instead of the market-fit product, we found that higher-level autonomous solutions were developed with customers in co-creation practices. Autonomous solutions open a variety of potential commercial setups with different ownership and revenue models. The setup that will prevail remains to be determined but, rather than merely developing and selling technology, the autonomous solutions provider must work to adopt a service dominant logic, providing maritime autonomous solutions as services. Additionally, leading the transition to maritime autonomous solutions demands the adoption of a forward-looking perspective that cherishes long-term growth over short-term profit.

“What we worked out after spending a huge amount of money is actually, we need to focus on what the customer actually needs, which is simple, and get to the real value for the customer. We have gone through that process ourselves, I would say painfully, but I think we are relatively pure now in how we develop the technology, there is a tip to sit there and ponder and do all these magical things but, at the end of the day, there is a business to run here and we, like I said, spend a lot of money that recognizes that you need to do the basics of the solutions to drive the value.” (VP business development, Shipcontrol).

In conclusion, the autonomous solutions provider experienced paradoxical tensions between an existing product mindset and the need to embrace a future-oriented service mindset. The paradox emerges because the mindsets represent opposing but necessary perspectives to the autonomous solutions provider’s organizational identity.

### Meso-level organizational paradoxes transitioning to autonomous solutions

The meso level focuses on paradoxes related to tensions arising from the interaction between ecosystem actors on forming a new autonomous solutions ecosystem. In the following paragraph, we elaborate on the two key paradoxes identified at this level: the ecosystem evolution paradox and the coopetition ecosystem paradox.

#### Ecosystem evolution paradox

Firstly, we find that moving to autonomous solutions provision creates an ***ecosystem evolution paradox***. With the formation of a new ecosystem, tensions arise between *creating new ecosystem partnerships* and *satisfying existing partners*. This is paradoxical for two reasons. First of all, ecosystem actors are stretched between maintaining their positions in the traditional ecosystem versus taking on a new position in the emerging ecosystem. Secondly, even when they assume the same role in both ecosystems, they are participating in competing ecosystems. The ecosystems are interrelated. The formation of a new ecosystem is an outgrowth of the traditional ecosystem because the actors rely on existing partnerships to build it, but the new ecosystem can be seen as a threat to the existing one. Transitioning the ecosystem is an evolutionary process, making the tensions persistent. The need for new collaboration was emphasized by several informants.

“We need to build relationships; we can’t do everything on our own.” (VP business concepts, Shipcontrol).

Materializing the value proposition of maritime autonomous solutions requires new competencies. The gap needs to be filled either by building the expertise internally within the existing ecosystem (leading to changed ecosystem roles) or by bringing in new actors with the competencies that the ecosystem requires. In taking a lead role in transforming the industry, the autonomous solutions provider worked continuously to identify its competency needs. It quickly realized that determining its specific requisites was an evolutionary learning process. The increasingly important role of connectivity and data processing in providing autonomous solutions led to the formation of new ecosystem partnerships with ITC companies. In addition to the importance of digital technology, maritime autonomy requires the operation of supporting service functions (SCC). In fact, one of the companies in our sample was a newly formed joint venture between a maritime technology provider and a ship management company. The aim in establishing the company was to provide autonomous services—a function that did not exist in the traditional ecosystem. It is not solely the need for competence that spurs change in ecosystem structures. So far, the demand for unmanned vessels has not come from traditional ship owners rather it has come from ship owners’ customers: namely, the goods owners. Curiously, with high-level autonomous solutions, we observe that the traditional chain of vessel ordering is reversed. Traditionally, ships are ordered from a shipyard, which gathers tenders for outfitting from technology providers. In the case of autonomous vessels, we found that the position is reversed with vessels being ordered directly from the technology provider, who in turn gathers tenders from shipyards.

“…and then it changed in a way, from that one orders a ship at a shipyard, which in turn goes out to pick up a bunch of subcontractors who supply different systems. So, the ship owner went to a technology supplier and wanted everything through them…” (Business development manager, Autosolution).

The *ecosystem evolution paradox* emerges because a company needs to strike a balance between participation in two ecosystems producing solutions that are in competition. The potential prevalence of autonomous solutions threatens an existing ecosystem structure, leaving some existing positions vulnerable to redundancy. On the contrary, the currently profitable existing ecosystem may itself impede adoption of maritime autonomy by providing cheap investment (low Capex) solutions. Therefore, while working to create new partnerships as the new maritime autonomy ecosystem evolves, the autonomous solutions provider must simultaneously satisfy its existing partnerships by maintaining its position as a technology provider to the traditional maritime ecosystem.

#### Coopetition ecosystem paradox

Secondly, we identified the ***coopetition ecosystem paradox*** as a related but distinct meso-level paradox. Tensions arise as technology providers need to collaborate closely with ecosystem partners to succeed in establishing maritime autonomy while, at the same time, competing for the ecosystem leadership position. This creates a situation where the ecosystem actors need to be both open and protective at the same time. Given the nested-ness of meso-level paradoxes, the tension will persist as long as the ecosystem continues to evolve, making it paradoxical.

“Also, we understand they wouldn’t have this open approach that [Shipcontrol] is having, that is to bring in competing OMs to offer value.” (VP business development, Shipcontrol).

While digitalization is at the technological core of autonomous solutions, an increased emphasis on services underpins its functional core. Both cores require closer and open collaboration with the ecosystem. To fully leverage digitalization, data need to flow easily between ecosystem actors. For instance, an autonomous vessel (ship owner) may want to share data via connectivity equipment (telecom) to the data platform (software company), which can then be further accessed by the autonomous service provider and other third-party software developers depending on customer needs. Fearing misuse or data going astray, some actors are reluctant to share with the ecosystem, leading to data silos that hamper autonomous solutions and prevent them from prevailing. This creates a subordinated data-sharing paradox, where actors need to both share and protect their data.

“When it comes to digitalization, we have a tendency to have a holistic view, but there are so many details we need to be very clear on, because data is crucial for the customer, and they don’t want to share data because it can be business or data critical. So, it is so many aspects and layers of digitalization of the shipping industry.” (VP digital product services, Shipcontrol).

The functional emphasis of autonomous solutions also drives the need for openness and transparency between ecosystem actors, breaking down organizational boundaries to deliver seamless services. Given the increasingly complex ecosystem associated with maritime autonomy, organizational boundaries have become blurred, leaving the collaborating actors vulnerable to role exploitation or marginalization. Thus, ecosystem actors must simultaneously collaborate with the ecosystem and work to improve their relative positions within the ecosystem.

### Macro-level organizational paradoxes transitioning to autonomous solutions

The macro-level paradoxes concern companies' relations with the highest aggregate-level entities such as markets and authorities. We identify two key paradoxes at this level: the customer interaction paradox and the regulatory and policy paradox. In the following paragraphs, we provide detailed accounts of both.

#### Regulatory and policy paradox

Firstly, the ***regulatory and policy paradox*** emerges from tensions between the autonomous solutions provider’s effort to *instigate new regulations* on the one hand and *cope with existing regulations* on the other. Technology providers need to change maritime regulations because they currently prevent commercialization of high-level autonomous solutions. Because the regulatory authorities are conservative and inherently reactive and regulations differ from nation to nation, changing regulations is a cumbersome process requiring persistence over the long term. Working to change regulations is an expensive process requiring collaborative efforts with uncertain outcomes that, if successful, will open doors for competitors. This creates a situation where all technology providers need settled regulations, but they must balance their efforts between pushing for regulatory change and coping with existing regulations. They are led into a “both-and” path, where they are required to work within existing regulations while trying to change them.

“If you want to change something, it will cost to find the technical solutions, regulations, business models, and get it better than it was before. And it usually costs more for the first movers that have to figure things out, both as a technical supplier, and to use it in operation.” (Sales director, Shipcontrol).

The commercial benefits of high-level autonomous solutions rest heavily on crew reduction, reduced crew costs, and increased load capacity. Regulations need to be changed to facilitate crew reduction so that high-level autonomous solutions can become commercially scalable. Relatedly, the commercial viability of shore control centers depends on efficient economies of scale, and so settled regulations are needed because it has still not been determined how many vessels one operator can serve simultaneously. Consequently, until regulations are firmly put in place, the commercial viability of high-level autonomous solutions remains uncertain, as the chairman of Autosolution’s board intimated:

“But it is many people talking about the technical risks of this, but that is the minor thing, for me the political risk is the biggest because we cannot see what the regulations will be in ten years. Every time I meet politicians, I try to tell them that we consider political risks as the biggest, the most difficult to handle in today’s market.” (Chairman, Autosolution).

The autonomous solutions provider acknowledged its own responsibility as leader to instigate regulatory change. Taking the lead in the change process is both risky and necessary. It could lead to first mover advantages because its solutions would likely be the first to accord with the new regulations. On the downside, it would have to bear the cost of instigation, while lowering the entry barriers for competitors by settling regulatory risk and possibly leading to first mover disadvantages. No single actor has the “gravity” to change regulations alone; therefore, technology providers collaborate with other ecosystem actors to demonstrate solution safety to local authorities and, in so doing, hope to influence the IMO.

“…And there is an IMO requirement that you should have a double-watch bridge. And to change things in IMO is quite challenging, and this double watched was challenged and piloted and there was some watch system.” (General manager, Marinecompany).

In working to change regulations, technology providers cope with existing regulations by looking for regulatory sweet spots where autonomous solutions could be deployed within existing regulations. Such sweet spots would include closed operation sites owned by customers where vessels can operate without encountering other vessels or personnel. Another way an autonomous solutions provider can cope with existing regulations is by aiming for a case-by-case approval for each vessel, rather than achieving permanent regulatory change.

“That is a long period of testing out in real life scenarios with crew onboard and gradually allowing the automated systems to take over more and more of the operation, until the legislative bodies feel satisfied that it is a safe operation, and they will grant approval for the vessel.” (Manager, Shipcontrol).

While collaboration with seafarers is crucial for successful implementation, the autonomous solutions that extend from monitoring to unmanned vessels create a fear of legislative pushbacks from seafarer organizations because autonomous solutions mean work surveillance and job losses. Therefore, our case companies emphasized the need to downplay the role of crew reduction when collaborating with seafarers while, at the same time, highlighting the benefits of the solutions to employers.

To summarize, the *regulatory and policy paradox* consists of persisting tensions between technology providers’ concurrent need to change regulations while coping with existing regulations. These tensions lead technology providers to embrace a “both-and” approach in dealing with regulations.

#### Customer interaction paradox

The second macro-level paradox identified is the ***customer interaction paradox.*** This paradox emerges from tensions between *adapting to novel customer segments* and *motivating traditional customers* to adopt autonomous solutions. The tensions consist of contradictory but interrelated elements. First of all, technology providers need to board the large traditional customer segment to successfully scale up their solutions. Paradoxically, while traditional customers are reluctant to change, technology providers work with a novel and somewhat competing customer segment, displaying economic benefits to traditional customers and safety to authorities. In trying to induce industrial change by moving to autonomous solutions, providers cannot choose to ignore either customer segment but must cater to both until traditional customer markets are boarded.

“We do see that customers who are in the maritime tradition want to do a gradual transformation, so we see a lot of the competitors in the monitoring aspect or the supervisor aspect, because they have a fleet and they want to better utilize that fleet, and we see many different instances of companies, but they are not taking over control from land, they are monitoring and supervising and giving support to the onboard crew.” (Product manager—Shipcontrol).

Traditional ship owners with an established fleet and a spreadsheet focus are well aware of the enormous legislative barriers in the way of maritime autonomy. Thus, they currently leverage second mover positions with a low investment profile. For example, they may be inclined to adopt lower-level autonomous solutions such as retrofit optimization/monitoring technology for their existing vessels. Even though technology providers depend on this segment for today's business, successful commercial scaling of autonomous solutions depends on convincing this segment to adopt higher-level autonomous solutions. Therefore, technology providers work to encourage the transition through gradual adaptations and synergic technologies that utilize the same technological core and service functions. Although interaction with traditional customers requires patience, technology providers stressed that today’s poor market cycle may in fact help to buy the time needed to transform the market.

“Many ship owners are dry docking vessels, which may be scrapped, and that can be an advantage for us. Potentially in a few years, there will be a demand for new vessels, and the cost of implementing these solutions are much cheaper on a new vessel than a retrofit. The advantages are much larger too. We are not ready yet, but in four or five years, if we get a year of new builds, then that is where we can take off. So even if this is bad for parts of our business with the current market situation as it is today. For remote and autonomous, it can be an advantage.” (Department manager, Shipcontrol).

Interaction with new customer types is indeed different. Unbounded by shipping’s historical legacy, new customer types have a more entrepreneurial approach to autonomy—less risk averse and more opportunity seeking—than traditional ship owners. Underscoring the paradox, this new customer segment consists of actors from the traditional customers’ own customer segment. Autonomous solutions help to remove huge entry barriers for non-maritime customers entering shipping. Seeking to enhance their logistic chain, autonomous solutions grant non-maritime actors the possibility of bypassing traditional ship owners and letting technology providers co-develop solutions tailored to meet the needs of end customers. Interacting with this customer segment, higher-level autonomy has shifted sales to higher levels in customer organizations. The autonomous solutions provider’s sales director emphasized how its sellers needed to change the focus to functional applications and rethink how customers could benefit from its solutions.

“I think we as sellers, we are not good, we, we can have some vessel and we know a lot of technology, but digitalization and applied digitalization to earn more or save more, we are not good enough at it. The sellers are not good enough, but I also think we hype digitalization without realizing how the customers actually are going to save on this or earn more if they have this.” (Sales director, Shipcontrol).

To conclude, the *customer interaction paradox* emerges from tensions between the simultaneous need to adapt to novel customer segments and to motivate traditional customers to adopt autonomous solutions. Despite their differences, both market segments are needed to form the future full-scale market for autonomous solutions. Working from both ends demands different approaches. Novel customers need to be enabled to bypass traditional ship owners, while traditional ship owners need to be motivated to adopt the solutions. Technology providers must endure these tensions until a substantial number of traditional customers come round to change, enabling commercial scaling.

### A multilevel paradox framework for autonomous solutions

Digital technologies have been crucial in our response to the current pandemic. They have enabled reduced physical contact while allowing us to work and socialize digitally (Zeng et al. 2020; López-Cabarcos et al. 2020). Similarly, in industrial settings, autonomous solutions offer a strengthening of supply chains from factory to delivery by reducing or even eliminating direct human contact. In this study, we adapted a multilevel framework for analyzing paradoxes (Garcia et al. 2019; Chistov et al. 2021). We identified six paradoxes inherent in the transition from technology provider to autonomous solutions provider, manifested at the micro, meso, and macro levels.

**Fig. 2:**
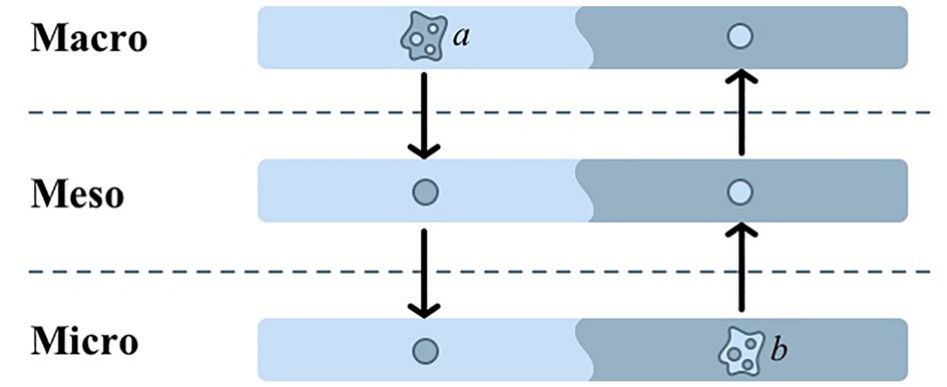
Paradox cascading effects

The multilevel paradox framework emphasizes the interaction between the paradoxes, meaning that events on one level can influence paradoxes across the aggregate levels (Sheep et al. 2017; Jarzabkowski et al. 2013; Andriopoulos and Lewis 2009; Smith and Lewis 2011). This may be the case if the paradoxes share underlying connections that create different challenging manifestations on different aggregate levels (Smith and Lewis 2011). By “zooming out” (Schad and Bansal 2018), we explore how events can influence a paradox by causing change to an underlying element on one level, which in turn influences paradoxes on other levels and creates upwards- (b in Fig. 2), or downwards- (a in Fig. 2) cascading effects across aggregate levels.

In the case of maritime autonomous solutions, we foresee such events as being related to changes in barriers or benefits that share communalities with some contradictory, interrelated, and persistent elements that generate paradoxical tensions. For example, in the *macro-level event* (a in Fig. 2) of a changed regulation, elements of the regulatory and policy paradox can be influenced directly. Such events may influence market structures affecting the customer interaction paradox, which in turn may cascade downwards, by changing the dynamics of the ecosystem role at the meso level, and induce a response in technology development at the micro level.

The cascading effect may just as well manifest itself upwards (b in Fig. 2). A *micro-level event* where a new technological solution has been developed can influence the technology development paradox. Such change in technology development can create a cascading effect affecting paradoxes across the aggregate levels. At the ecosystem level, such changes might, for instance, alter the need for competence or affect the level of competition between ecosystem actors, thereby influencing the ecosystem evolution paradox or the coopetition ecosystem paradox. The macro level may in turn be affected, prompting a market reaction or altering the need for regulations or policy standards, with consequent effects on the customer interaction paradox and the regulatory and policy paradox.

While our study context was confined to the shipping industry, we expect our findings to resonate with advanced digital servitization processes in other industries. That is to say, the elements from which the paradoxes emerge are not limited to the shipping industry. Shifting from technology provider to autonomous solutions provider requires internal changes in technology development and organizational identity, as well as changes in relationships with external entities. The materialization of an autonomous solutions value proposition simultaneously drives change and is driven by changes in the ecosystem structure (Thomson et al. 2021). On the meso level, common elements regarding the ecosystem position would be present in most industry settings. Our findings of the *coopetition ecosystem paradox* and the *ecosystem evolution paradox* identify situations where the fit alignment goal of contingency theory may never be completely achieved (Donaldson 2001). In such situations, developing coping strategies would be a better response. Although not the main purpose of this study, we witnessed some coping strategies: on the micro level, the technology providers worked to address technology development paradox by developing synergies between solutions. On the meso level, the case companies have responded by creating joint functions working across firm boundaries while, on the macro level, they have been working primarily to influence customers and regulatory authorities locally, before broadening the scope.

As autonomous solutions generally benefit from reduced human interaction, the need to have settled regulations (Hussain and Zeadally 2019; Ringbom 2019) and the requirement to bring about change in traditional business models are elements common across industry settings (Porter and Heppelmann 2014; Thomson et al. 2021). Therefore, our context-specific findings should hold some degree of external validity and may well extend to other digitalization processes (Parida et al. 2019). In a very wide sense, the identified paradoxes hold elements that can be linked to tension between past and future as digital servitization toward autonomous solutions is a process that requires changing traditional business models at an industrial level (Gaiardelli et al. 2021; Thomson et al. 2021). For instance, the organizational identity paradox, the technology development paradox, and the customer interaction paradox share underlying elements between past and future (manifested at their respective levels), as traditional value offerings change from product plus after sales services toward a future full-value solution (Gaiardelli et al. 2021). So too, the ecosystem evolution paradox and the coopetition paradox hold elements whose tensions can be linked to the value creation process moving from a traditional transaction-based relationship with clear boundaries, toward a future sharing and symbiotic relationship (Gaiardelli et al. 2021).

## Discussion and conclusion

### Theoretical contribution

This study makes three theoretical contributions. Firstly, because digital servitization processes are intrinsically complex involving a multitude of actors, there is a need for a multilevel framework to understand its paradoxical nature (Kohtamäki et al. 2020). To address this, we adapted a multilevel framework from open innovation to analyze paradoxes of advanced digital servitization (Garcia et al. 2019; Chistov et al. 2021). Thus, we were able to identify and describe six paradoxes inherent in the shift to autonomous solutions provision, and thereby showing that technology providers are in fact facing paradoxes, rather than dilemmas. Our findings on the micro-level *organizational identity paradox* and the *technology development paradox* corroborate and extend previous studies of paradoxes in servitization (Kohtamäki et al. 2020). The technology development paradox echoes the archetype paradox of exploration and exploitation (Andriopoulos and Lewis 2009), as technology providers need to simultaneously pursue both as they move toward autonomous solution provision. The organizational identity paradox adds to the discussion of mindset and culture present as an overarching theme in the digital servitization literature (Tronvoll et al. 2020), by discussing how advanced digital servitization triggers the need to incorporate a future service mind-set into an existing product mind-set spurring paradoxical tensions in both front-end and back-end of the technology providers organization.

Digitalization drives changes in business ecosystems, by heightening the need for competence and resource sharing among ecosystem actors (Parida et al. 2019; Thomson et al. 2021; Kohtamäki et al. 2019). Our findings of ecosystem evolution paradox demonstrate how technology providers are faced with tensions between satisfying traditional partnerships to maintain their ecosystem position while creating new ecosystem partners and changing ecosystem roles. Furthermore, our finding of the coopetition ecosystem paradox adds to digital servitization literature by showing how coopetition paradox is intrinsic to the advanced digital servitization process (Raza-Ullah et al. 2014; Bengtsson and Kock 2014).

On the macro level, we identify the *customer interaction paradox* and the *regulatory and policy paradox* describing how technology providers face paradoxical tensions in interacting with macro-level entities*.* The macro-level paradoxes demonstrate how the intersection between opportunities and barriers for autonomous solutions spur paradoxical tensions, where technology providers need to weigh their efforts to drive necessary macro-level changes.

Secondly, our data suggest that moving to advanced digital servitization by providing autonomous solutions increases complexity. A multitude of stakeholders constantly drive changes, promoting influencing events on the meso and macro levels and, consequently, making the external paradoxes more dominant. Thus, by identifying and describing the paradoxes through a multilevel framework, we provide insights into why technology providers must actively embrace the meso- and macro-level perspectives in addition to their internal standpoints. We thereby expand the current knowledge of paradoxes in servitization and digitalization to include external perspectives (Kohtamäki et al. 2020; Smith and Beretta 2021).

Thirdly, adding to the complexity underpinning the paradoxes at each aggregate level, the paradoxes themselves interlinked across the levels. This is a problem for companies working to cope with paradoxes because their practices might be directed to paradoxes on one level but are influenced on another level that is increasingly beyond their control. Nested relationships among paradoxes are well documented in the literature (Smith and Lewis 2011; Jarzabkowski et al. 2013; Sheep et al. 2017). We contribute to knowledge of paradoxes in digital servitization by provided guiding examples from our empirical context to show how changes in the underlying elements of a paradox on one level may trigger cascading effects that influence paradoxes across levels (Smith and Lewis 2011).

### Managerial contribution

Because the digital servitization process toward autonomous solutions requires ecosystem leaders to actively drive the process, managers should be aware of the paradoxical tensions inherent in this process. As paradoxes may be latent (Smith and Lewis 2011), or even present without being perceived (Schad and Bansal 2018), our findings should be of interest to managers leading advanced digital servitization processes. On the strength of our analysis, we encourage managers to develop coping strategies by embracing the paradoxical elements rather than trying to choose between them. For example, the case company coped with the micro-level *technology development paradox* by creating spin-off products and synergies between solutions, partly reconciling explorative autonomous technology development with adaptive technology development. Our findings of the organizational identity paradox inform managers that to succeed with digital servitization, they need to cherish both a future service mind-set and a product mind-set. Kohtamäki et al. (2020) suggest that managers can cope with this paradox through strategic work to bridge product and service thinking and through development programs to induce shared understanding. On the meso level, the coopetition ecosystem paradox highlights the importance of working toward ecosystem alignment. Managers leading digital servitization processes should seek to relieve tensions between ecosystem actors by creating an ecosystem strategy that aligns actors’ goals and incentives (Adner 2017). As ecosystems are evolving structures, it is likely that the alignment will need to be continuously renegotiated. The multilevel framework highlights how complexity rises in line with the aggregate level. Our finding of the macro-level regulatory and policy paradox should alert managers that their efforts to instigate regulatory changes, at a high cost, may ultimately open doors to competitors. Thus, managers are encouraged to balance their relative contributions to change against their expected outcomes. Moreover, as paradoxes are nested, managers leading digital servitization processes should be aware possible of cascading effects when trying to cope with paradoxes. Measures taken to deal with a paradox on one level may affect paradoxes at other levels. For example, actions taken to deal with the customer interaction paradox on the macro level may strengthen or weaken the salience of the technology development paradox on the micro level.

Our findings inform decision makers who are considering embarking on similar industrial transitions. Though paradoxes cannot be avoided individually, deciding whether to engage in a digital servitization process leading to autonomous solutions is a dilemma (Christensen 1997). Therefore, we encourage managers to carefully consider the paradoxes identified before making such decisions. After all, our findings may equally help to explain why many digitalization and servitization projects fail to yield the expected returns (Parida et al. 2019; Gebauer et al. 2005; Hyun and Kim 2021).

### Limitations and future research

We acknowledge that this study has several limitations. Firstly, as the intent of this study is to identify paradoxes inherent in the shift to autonomous solutions, it does not explicitly identify coping practices. Future studies could address how companies cope with the identified paradoxes, especially those on the meso and macro levels. Secondly, due to the in-depth single-case data collection, the six paradoxes identified may be case specific. Thus, we encourage future researchers to adopt a multiple-case approach, collecting data from multiple industries. Collecting data from the broader ecosystem would also help corroborate the validity of higher-level paradoxes and enable cross-case analysis. Thirdly, as the list of paradoxes identified is not exhaustive, further research may reveal new paradoxes. Future studies could apply quantitative methods to test the implications and severity of these paradoxes on management indicators such as firm performance.

## References

[CR1] Adner R (2017). Ecosystem as structure: an actionable construct for strategy. J Manag.

[CR2] Al-Omoush KS, Simón-Moya V, Sendra-García J (2020). The impact of social capital and collaborative knowledge creation on e-business proactiveness and organizational agility in responding to the COVID-19 crisis. J Innov Knowl.

[CR3] Andriopoulos C, Lewis MW (2009). Exploitation-exploration tensions and organizational ambidexterity: managing paradoxes of innovation. Organ Sci.

[CR4] Baines TS, Lightfoot HW, Benedettini O, Kay JM (2009). The servitization of manufacturing. J Manuf Technol Manag.

[CR5] Baron S, Patterson A, Maull R, Warnaby G (2018). Feed people first: a service ecosystem perspective on innovative food waste reduction. J Serv Res.

[CR6] Bengtsson M, Kock S (2014). Coopetition—quo vadis? Past accomplishments and future challenges. Ind Mark Manage.

[CR7] Braun V, Clarke V (2006). Using thematic analysis in psychology. Qual Res Psychol.

[CR8] Cenamor J, Rönnberg Sjödin D, Parida V (2017). Adopting a platform approach in servitization: leveraging the value of digitalization. Int J Prod Econ.

[CR9] Chiang A-H, Trimi S (2020). Impacts of service robots on service quality. Serv Bus.

[CR10] Chistov V, Aramburu N, Carrillo-Hermosilla J (2021). Open eco-innovation: a bibliometric review of emerging research. J Clean Prod.

[CR11] Christensen CM (1997). The innovator’s dilemma: when new technologies cause great firms to fail.

[CR12] Corbin J, Strauss A (2015). Basics of qualitative research: techniques and procedures for developing grounded theory.

[CR13] de Bellis E, Johar GV (2020). Autonomous shopping systems: identifying and overcoming barriers to consumer adoption. J Retail.

[CR14] Donaldson L (2001). The contingency theory of organizations.

[CR15] Dyer WG, Wilkins AL (1991). Better stories, not better constructs, to generate better theory: a rejoinder to Eisenhardt. Acad Manag Rev.

[CR16] FinancesOnline (2020) 72 Vital digital transformation statistics: 2021/2022 spending, adoption, analysis and data. https://financesonline.com/digital-transformation-statistics/. Accessed 4 Aug 2021

[CR17] Frow P, McColl-Kennedy JR, Hilton T, Davidson A, Payne A, Brozovic D (2014). Value propositions: a service ecosystems perspective. Mark Theory.

[CR18] Gaiardelli P, Pezzotta G, Rondini A, Romero D, Jarrahi F, Bertoni M, Wiesner S, Wuest T, Larsson T, Zaki M, Jussen P, Boucher X, Bigdeli AZ, Cavalieri S (2021). Product-service systems evolution in the era of Industry 4.0. Serv Bus.

[CR19] Garcia R, Wigger K, Hermann RR (2019). Challenges of creating and capturing value in open eco-innovation: evidence from the maritime industry in Denmark. J Clean Prod.

[CR20] Gebauer H, Fleisch E, Friedli T (2005). Overcoming the service paradox in manufacturing companies. Eur Manag J.

[CR21] Gebauer H, Paiola M, Saccani N, Rapaccini M (2021). Digital servitization: crossing the perspectives of digitization and servitization. Ind Mark Manage.

[CR22] Ghaderi H (2019). Autonomous technologies in short sea shipping: trends, feasibility and implications. Transp Rev.

[CR23] Ghazinoory S, Nasri S, Ameri F, Montazer GA, Shayan A (2020). Why do we need problem-oriented innovation system (PIS) for solving macro-level societal problems. Technol Forecast Soc Chang.

[CR24] Hussain R, Zeadally S (2019). Autonomous cars: research results, issues, and future challenges. IEEE Commun Surv Tutor.

[CR25] Hyun M, Kim J (2021). Challenge or opportunity? A case of tire rental servitization from financial and channel perspectives. Serv Bus.

[CR26] Jarzabkowski P, Lê JK, Van de Ven AH (2013). Responding to competing strategic demands: How organizing, belonging, and performing paradoxes coevolve. Strateg Organ.

[CR27] Kamalaldin A, Sjödin D, Hullova D, Parida V (2021). Configuring ecosystem strategies for digitally enabled process innovation: a framework for equipment suppliers in the process industries. Technovation.

[CR28] Kiefer CP, del Río P, Carrillo-Hermosilla J (2021). On the contribution of eco-innovation features to a circular economy: a microlevel quantitative approach. Bus Strateg Environ.

[CR29] Kim Y-J, Lee S-G, Trimi S (2021). Industrial linkage and spillover effects of the logistics service industry: an input–output analysis. Serv Bus.

[CR30] Kohtamäki M, Parida V, Oghazi P, Gebauer H, Baines T (2019). Digital servitization business models in ecosystems: a theory of the firm. J Bus Res.

[CR31] Kohtamäki M, Einola S, Rabetino R (2020). Exploring servitization through the paradox lens: coping practices in servitization. Int J Prod Econ.

[CR32] Kraus S, Clauss T, Breier M, Gast J, Zardini A, Tiberius V (2020). The economics of COVID-19: initial empirical evidence on how family firms in five European countries cope with the corona crisis. Int J Entrep Behav Res.

[CR33] Lerch C, Gotsch M (2015). Digitalized product-service systems in manufacturing firms: a case study analysis. Res Technol Manag.

[CR34] Leung E, Paolacci G, Puntoni S (2018). Man versus machine: resisting automation in identity-based consumer behavior. J Mark Res.

[CR35] Levinson M (2016). The box: how the shipping container made the world smaller and the world economy bigger.

[CR65] Linde L, Sjödin D, Parida V, Gebauer H (2021). Evaluation of Digital Business Model Opportunities. Res-Technol Manag.

[CR36] López-Cabarcos MÁ, Ribeiro-Soriano D, Piñeiro-Chousa J (2020). All that glitters is not gold. The rise of gaming in the COVID-19 pandemic. J Innov Knowl.

[CR37] Lorange P (2009). Shipping strategy: innovating for success.

[CR38] Markard J, Truffer B (2008). Technological innovation systems and the multi-level perspective: towards an integrated framework. Res Policy.

[CR39] Miles MB, Huberman AM, Saldaña J (2014). Qualitative data analysis: a methods sourcebook.

[CR40] Munim ZH (2019). Autonomous ships: a review, innovative applications and future maritime business models. Supply Chain Forum Inter J.

[CR41] Nambisan S, Lyytinen K, Majchrzak A, Song M (2017) Digital Innovation Management: Reinventing innovation management research in a digital world. Mis Quarterly 41 (1)

[CR42] Paiola M, Gebauer H (2020). Internet of things technologies, digital servitization and business model innovation in BtoB manufacturing firms. Ind Mark Manage.

[CR43] Parida V, Sjödin D, Reim W (2019). Reviewing literature on digitalization, business model innovation, and sustainable industry: Past achievements and future promises. Sustainability.

[CR44] Patrıcio L, Fisk RP, Cunha JF, Constantine L (2011). Multilevel service design: from customer value constellation to service experience blueprinting. J Serv Res.

[CR45] Patton MQ (1990). Qualitative evaluation and research methods.

[CR46] Porter ME, Heppelmann JE (2014). How smart, connected products are transforming competition. Harv Bus Rev.

[CR47] Rapaccini M, Saccani N, Kowalkowski C, Paiola M, Adrodegari F (2020). Navigating disruptive crises through service-led growth: the impact of COVID-19 on Italian manufacturing firms. Ind Mark Manage.

[CR48] Raza-Ullah T, Bengtsson M, Kock S (2014). The coopetition paradox and tension in coopetition at multiple levels. Ind Mark Manage.

[CR49] Rijsdijk SA, Hultink EJ (2003). Honey, have you seen our hamster? Consumer evaluations of autonomous domestic products. J Prod Innov Manag.

[CR50] Ringbom H (2019). Regulating autonomous ships—concepts, challenges and precedents. Ocean Dev Inter Law.

[CR51] Rødseth J (2017) From concept to reality: unmanned merchant ship research in Norway. In 2017 IEEE underwater technology (UT), 21–24 Feb, pp 1–10. 10.1109/UT.2017.7890328

[CR52] Schad J, Bansal P (2018). Seeing the forest and the trees: how a systems perspective informs paradox research. J Manage Stud.

[CR53] Sheep ML, Fairhurst GT, Khazanchi S (2017). Knots in the discourse of innovation: investigating multiple tensions in a reacquired spin-off. Organ Stud.

[CR64] Sjödin D, Parida V, Palmié M, Wincent J (2021) How AI capabilities enable business model innovation: Scaling AI through co-evolutionary processes and feedback loops. J Bus Res 134574–134587. 10.1016/j.jbusres.2021.05.009

[CR54] Sklyar A, Kowalkowski C, Tronvoll B, Sörhammar D (2019). Organizing for digital servitization: a service ecosystem perspective. J Bus Res.

[CR55] Smith P, Beretta M (2021). The gordian knot of practicing digital transformation: coping with emergent paradoxes in ambidextrous organizing structures. J Prod Innov Manag.

[CR56] Smith WK, Lewis MW (2011). Toward a theory of paradox: a dynamic equilibrium model of organizing. Acad Manag Rev.

[CR57] Teixeira J, Patrício L, Nunes NJ, Nóbrega L, Fisk RP, Constantine L (2012). Customer experience modeling: from customer experience to service design. J Service Manage.

[CR58] Thomson L, Kamalaldin A, Sjödin D, Parida V (2021). A maturity framework for autonomous solutions in manufacturing firms: The interplay of technology, ecosystem, and business model. Inter Entrepreneurship Manage J.

[CR59] Tronvoll B, Sklyar A, Sörhammar D, Kowalkowski C (2020). Transformational shifts through digital servitization. Ind Mark Manage.

[CR60] Williamson OE (1979). Transaction-cost economics: the governance of contractual relations. J Law Econ.

[CR61] Xie X, Zang Z, Ponzoa JM (2020). The information impact of network media, the psychological reaction to the COVID-19 pandemic, and online knowledge acquisition: evidence from Chinese college students. J Innov Knowl.

[CR62] Yin RK (2014). Case study research design and methods.

[CR63] Zeng Z, Chen P-J, Lew AA (2020). From high-touch to high-tech: COVID-19 drives robotics adoption. Tour Geogr.

